# Endozoochory of *Chrysobalanus icaco* (Cocoplum) by *Gopherus polyphemus* (Gopher Tortoise) facilitates rapid germination and colonization in a suburban nature preserve

**DOI:** 10.1093/aobpla/plaa024

**Published:** 2020-06-19

**Authors:** Carolyn J Hanish, Sebastian Velez, Jon A Moore, Corey Devin Anderson

**Affiliations:** 1 Department of Biological Sciences, Florida Atlantic University, Boca Raton, FL, USA; 2 School of Veterinary Medicine and Biomedical Sciences, University of Nebraska-Lincoln; Lincoln, NE, USA; 3 NOAA Office of Law Enforcement, Silver Spring, MD, USA; 4 Wilkes Honors College, Florida Atlantic University, Jupiter, FL, USA; 5 Department of Biology, Valdosta State University, Valdosta, GA, USA

**Keywords:** Burrow, Chyrsobalanaceae, point pattern analysis, point process model, reptile seed dispersal, scarification, time to event analysis

## Abstract

Some large-seeded plants lack effective seed dispersal agents when they are introduced as ornamental plants to new areas, but can rapidly colonize a landscape if seed dispersal functions are restored. We examined whether *Gopherus polyphemus* (Gopher Tortoise) facilitated the spread of *Chrysobalanus icaco* (Cocoplum; Chrysobalanaceae) over a 14-year period in a suburban nature preserve (in Jupiter, FL, USA) by: (i) comparing germination patterns among gut-passed, hand-depulped and whole fruit treatments, and (ii) testing hypotheses about environmental predictors of the spatial distribution of *C. icaco*, including information about *G. polyphemus* movement pathways and burrow locations. While we did not find a significant difference in the total proportion of *C. icaco* seeds that germinated in each treatment, time to event analysis revealed that seeds that were found in faeces germinated significantly earlier than seeds that were hand-depulped or that were planted as whole fruits, supporting a lone scarification effect. Point process modeling revealed that the density of *C. icaco* bushes was higher near *G. polyphemus* movement pathways and was lower inside *Serenoa repens* (Saw Palmetto) patches, supporting a positive effect of tortoise movement patterns on plant distributions. The density of *C. icaco* increased from west to east, consistent with westward dispersal from the four founder bushes on the east side of the study area. After removal of outliers, we also detected a negative association between *C. icaco* spatial density and *G. polyphemus* burrow density that was presumably explained by the fact that seeds defecated deep within burrows were unlikely to germinate and establish without secondary movement. The results suggest that *G. polyphemus* contributed to the rapid dispersal of *C. icaco* by scatter dispersal of seeds (via faeces) in areas where tortoises were active and that movement pathways provided suitable conditions for colonization. The spread of *C. icaco* by *G. polyphemus* over a relatively short period of time provides a valuable window into the earliest stages of the colonization process and further supports the role of Chelonians as effective seed dispersal agents for large-seeded plants.

## Teaser

Plants that produce large, fleshy fruits are sometimes introduced to new areas for ornamental purposes, but may not spread further due to the absence of animals that are capable of dispersing their seeds. We examined a case where four cocoplum bushes were introduced to a suburban gopher tortoise refuge (in Jupiter, FL, USA) for aesthetic purposes and subsequently spread throughout the area, over a 14-year period. We found evidence that the gopher tortoise likely contributed to cocoplum expansion, by accelerating seed germination and by defecating seeds in areas, such as trails, that provided suitable locations for cocoplum establishment.

## Introduction

How seed dispersal by animals affects the spatial distribution of plants is a major topic in the ecological literature due to its fundamental importance to the structure of almost all natural ecosystems (Nathan and Muller-Landau 2000; Wang and Smith 2002; Schupp *et al.* 2010). Seeds can be transported by animals externally (= epizoochory) or ingested and later excreted (= endozoochory; Traveset *et al.* 2007). In frugivorous endozoochory, the animal receives a nutritional reward when ingesting the fruit, and the seeds benefit by travelling away from their parent’s shadow (Janzen 1970, 1971; Connell 1971), potentially allowing them to germinate in new areas with favourable conditions (Howe and Smallwood 1982; Howe 1989).

Ingestion of seeds by animals can have varying effects on seed germination (Traveset *et al.* 2007). Some animals may damage seeds during mastication and gut passage resulting in seed death or reduced viability (Herrera 2002; Traveset and Verdú 2002). In other cases, gut passage may be neutral or it may enhance the speed of germination or the total proportion of germinated seeds by removal of germination inhibitors in pulp that surrounds seeds (Yagihashi *et al.* 1998; Robertson *et al.* 2006), by promoting scarification of the seed coat that enhances imbibition of water (Cipollini and Levey 1997; Traveset 1998) or by providing a source of fertilizer upon defecation (Dinerstein and Wemmer 1988; Traveset *et al.* 2001a). The effect of ingestion on seed germination is also affected by seed characteristics. The size, thickness or permeability of the seed coat can contribute to the success or failure of gut passage (Traveset and Verdú 2002; Milotić and Hoffmann 2016). Different combinations of seed characteristics and animal gastrointestinal properties can make the outcome of endozoochory dependent on the individual plant and/or animal species (Izhaki and Safriel 1990; Traveset *et al.* 2001b, 2007; Fedriani and Delibes 2009).

Most vertebrates have been identified as seed dispersers, though the majority of well-known plant dispersers are birds and mammals (Stiles 2000). In the present day, reptiles are rarely the primary herbivore in terrestrial ecosystems, but many lizards, turtles and tortoises are important seed dispersal agents in insular and arid systems (van der Pijl 1982; Valido and Oleson 2007; Falcón *et al.* 2020). For some plants, endozoochory by Chelonians (turtles and tortoises) may provide distinct advantages over other animals, based on their feeding characteristics. Chelonians lack teeth and have a propensity to swallow their food whole; for this reason, they are less likely to damage seeds during mastication and ingestion (Moll and Jansen 1995; Birkhead *et al.* 2005; Jerozolimski *et al.* 2009). These characteristics may make Chelonians particularly important dispersers for many plants with large seeds (Birkhead *et al.* 2005; Jerozolimski *et al.* 2009). There has been a boom in interest in the use of tortoises as seed disperser substitutes (especially on oceanic islands) to restore ecosystem functions that were lost due to the extinction of large herbivores, including native tortoise species (Hansen *et al.* 2008, 2010; Kaiser-Bunbury *et al.* 2010; Griffiths *et al.* 2011). There has also been increasing concern about the potential for tortoises to disperse introduced plant species and, conversely, on the impact of introduced tortoise species on native plant communities (Heleno *et al.* 2013; Blake *et al.* 2015; Ellis-Soto *et al.* 2017).

The plant species of interest in our study is *Chrysobalanus icaco* (Cocoplum), a shrub native to South Florida, USA, though its range extends south into the Caribbean, Central and South America, and tropical western Africa (Francis 2004). *Chrysobalanus icaco* is known to grow in thickets on sandy soils and singly on rock outcrops, and is often used in South Florida as an ornamental plant (Francis 2004; Daley and Zimmerman 2007). The leaves and bark of *C. icaco* have a history of use in traditional medicine, and its fruits are consumed by both humans and wildlife (Francis 2004). The fruit of *C. icaco* ranges from deep purple to white, depending on variety, and is a drupe with a soft and slightly sweet flesh surrounding the endocarp, that is generally referred to as the stone or pit which resembles a longitudinally ridged teardrop and has a white seed protected inside. The fruits ripen and fall to the ground passively, and can drift long distances by water; when they are found on shorelines, only the stone/pit containing the seed remains. Although many animals have been implicated as potential dispersal agents of *C. icaco* (Francis 2004), the size and weight of the fruit make it unlikely to be dispersed by frugivores with gape limitations for *C. icaco*, such as small birds (Wheelwright 1985). Conversely, these characteristics make *C. icaco* seeds ideal candidates for dispersal by large-bodied lizards (such as Iguanids) and Chelonians that are capable of swallowing the fruit whole.

*Gopherus polyphemus* (Gopher Tortoise) is a North American tortoise found in the south-eastern USA. Its range overlaps with that of *C. icaco* in South Florida, USA, where the tortoise is associated with scrub forests, flatwoods and xeric (dry) upland ridges (Castellón *et al.* 2018, 2020). Because it excavates burrows in the ground that are inhabited by a multitude of other species, *G. polyphemus* is widely considered to be a keystone species (Franz 1986; Jackson and Milstrey 1989; Lips 1991; Kent *et al.* 1997). The turning of soil at burrow entrances by the tortoise can also affect plant establishment, by returning leached nutrients and seeds deposited within the burrow back to the surface, and by providing a disturbed surface for plant colonization (Kaczor and Hartnett 1990). While *G. polyphemus* is best known for the effects of its burrowing, its grazing is also ecologically important due to the wide range of plants that it consumes (MacDonald and Mushinsky 1988; Mushinsky *et al.* 2003; Birkhead *et al.* 2005; Richardson and Stiling 2019). For example, Ashton and Ashton (2008) list 225 genera of plants and 400–500 plant species that are consumed by the *G. polyphemus*. They are known to swallow large-seeded fruits (such as *Licania michauxii*, Gopher Apple; Birkhead *et al.* 2005) whole and pass seeds intact.

**Figure 1.**
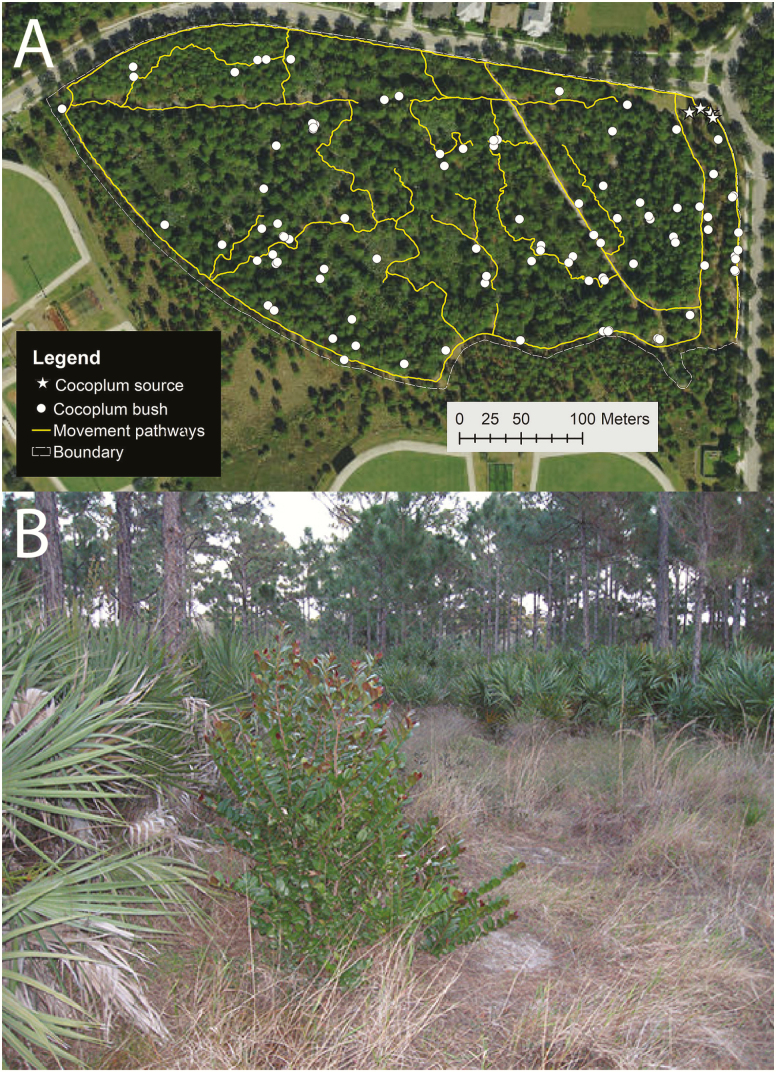
(A) Magnification of the 9.27-ha study area (grey dashed line) within the Abacoa Greenway near Jupiter, FL, USA. The original four *Chrysobalanus icaco* (Cocoplum) bushes are represented by white stars and descendent *C. icaco* bushes are represented by white dots. The yellow lines represent known *Gopherus polyphemus* movement pathways. (B) Photograph of the field site showing a single, subadult *C. icaco* bush along a wide movement pathway covered primarily by *Paspalum notatum*. *Serenoa repens* and *Pinus elliottii* are also visible in the foreground and background.

Our study is based on a unique case where a small number of *C. icaco* bushes were introduced as ornamentals to a suburban nature preserve in Jupiter, FL, USA (Fig. 1) containing a substantial population of *G. polyphemus*. The *C. icaco* bushes on site have a known introduction location and date. Four subadult plants (~1 m in height) were planted in early spring of 2004 to block the view of a cement drainage culvert in the northeast corner of the site; they began to produce fruit in late 2006. By 2017, the *C. icaco* population had expanded (to 99 bushes) across the upland portion of the study area (Fig. 1A). We examined how *G. polyphemus* may have facilitated the spread of *C. icaco* in the nature preserve in two ways. First, using *C. icaco* seeds found in *G. polyphemus* faeces and fruits collected in the field, we conducted experiments to tease apart the effects of scarification and deinhibition on *C. icaco* germination, and we used time to event analysis to test for differences in germination proportions during the time of the experiment. Second, we used point pattern analysis (Wiegand and Moloney 2004; Baddeley *et al.* 2015) to examine the spatial distribution of *C. icaco* bushes within our study area. We tested whether the number of *C. icaco* plants in different areas was dependent on certain environmental covariates (including information about tortoise movement pathways and burrow locations) and if, after correction for spatial dependency, there was evidence of clustering due to restricted seed dispersal from parental plants or via clump dispersal of seeds in scats.

## Materials and Methods

### Study site

The study site for this project was located in a 13.45-ha (~33 acre) section of a greenway within the community of Abacoa, a residential development in Jupiter, FL, USA (26.90°N, 80.11°W; Fig. 1). The site is surrounded by a perimeter chain link fence and acts as a *G. polyphemus* preserve that has been monitored closely since 2001 (Wetterer and Moore 2005). The central habitat is an elevated upland ~9.27 ha (~23 acre) in size. A mowed pathway blanketed by *Paspalum notatum* (Bahiagrass) and *Richardia* spp. (Pusley) encircles the central area, and two straight line paths crossing the northern and eastern sections are also occasionally maintained and cover an old (prior to 1996) cleared cattle fence line and a buried pipeline (circa 1999; Wetterer and Moore 2005). The upland closely resembles a xeric or scrubby flatwood habitat with sparse canopy consisting of mature *Pinus elliottii* (Slash Pine), *Quercus* spp. (Scrubby Oaks) and a thick understory of *Serenoa repens* (Saw Palmetto). The perimeter fence acts as a barrier to emigration of *G. polyphemus*, though on rare occasions new individuals are introduced (as waif tortoises) and some individuals manage to escape. During this study, the estimated population of *G. polyphemus* on this site was ~110 individuals (J. A. Moore, pers. obs.).

*Gopherus polyphemus* burrows are primarily located along the upland wooded range, but were occasionally found in the catchment basin that borders the southern and western portions of the range. The basin is lower in elevation and acts as a water retention site for the nearby housing development, though it rarely held standing water outside of intense flooding events. Because of the thick understory of *S. repens* in the upland range, tortoises used the mowed pathways, as well as narrow tortoise pathways throughout the interior vegetation that have been maintained by high tortoise activity.

### Seed collection and planting

Seed and fruit collection were conducted in the upland section of the greenway. All gut-passed seeds found in tortoise faeces came from this field site, as well as all whole, ripe fruits. Faecal sampling occurred at the study site on a weekly basis from 3 August to 1 November 2016. Fresh faecal samples (*n* = 34) were collected opportunistically from throughout the site. A faecal sample was collected if the seeds remained inside and the faeces retained some moisture; if the faeces had lost shape integrity or was missing a part of the sample, it was disregarded. When *G. polyphemus* faeces were found, field dissection was performed to determine whether *C. icaco* seeds were present. In the field, faecal samples were dissected on portable trays to preserve as much of the original sample as possible. Each faecal sample was stored in its own paper bag to dry. As each sample dried, seed and faeces were separated and stored in dry conditions until they were sorted into their individual trays. A total of 108 *C. icaco* seeds were recovered from the 34 faeces. The number of seeds per faeces ranged from 1 to 20 **[see****Supporting Information—Fig. S1****]**; the distribution was right-skewed (mode = 1; median = 3; max = 20). Additional collection of ripe fruit occurred during the fruit-bearing season. Because fruits were at varying stages of desiccation when encountered, all collected fruits were stored in the same controlled environment until the experiment commenced. Fruits were then assigned to one of two treatments to compare to the gut-passed seeds: (i) hand depulped using hemostat forceps (= hand depulped), or (ii) planted as is, with the exocarp and mesocarp intact around the endocarp and seed (= whole fruit).

In total, 108 gut-passed seeds, 104 hand-depulped seeds and 106 whole fruit seeds were prepared and organized into labelled cells in five trays. Each tray contained 72 cells arranged in a 6 × 12 grid, where each cell was 3.8 × 3.8 cm in surface area and 5.7 cm deep. To plant the 318 seeds, we used four trays completely and half of the fifth tray to total 324 cells, where the residual six cells had all conditions held constant, but no seeds were planted in them. Each seedling cell was assigned a pseudorandom number between 1 and 324 using a Fisher–Yates shuffle algorithm. This ensured that each tray of seeds had a pseudorandom pattern and that no seedling neighbours were intentionally grouped by treatment. Because the seed trials were implemented indoors in a laboratory setting, seed trays were positioned on a heat mat with a thermostat controller and timer in order to provide a gradient between day 32 °C and night 21 °C temperatures for 12-h day and 12-h night cycles throughout the experiment.

On 27 January 2017, all *C. icaco* seeds were planted in their respective cells using 8–10 g of Pro-Mix HP Mycorrhizae soil, which was chosen for its high-porosity drainage and for reproducibility in experiment design. Each seed was buried within 1 cm of the surface to reproduce natural conditions of sowing after faecal deposit and leaf litter application. Each cell received water until soil saturation occurred, without standing water at the surface. The seedling cells were rewatered when the soil was no longer visibly saturated. Each tray was rotated every 3 days on the shelving unit to control for microclimate bias. Observations were made daily after the first germination occurred on 14 March 2017. For the purposes of this experiment, germination was considered successful if any portion of the plant was observable from the surface, most often the hypocotyl or radicle of the germinating seed (with consistent watering, some seeds drifted to the soil surface, exposing their seed coats and allowing the radicle to be observed upon germination). The study concluded after 165 days on 25 August 2017. At this point, if no portion of the seedling penetrated the seed coat, a seed was considered to not have germinated within the time frame of the experiment and was right censored for analysis (McNair *et al.* 2012).

### Spatial data collection

Geospatial data were collected between 5 December 2016 and 7 July 2017 with a Trimble Juno 3 with a ProXH Global Navigation Satellite System (GNSS) receiver. The Trimble hardware used Terrasync software for efficient field data measurements and collection, as well as post-processing (i.e. differential correction using nearby base stations; CORS, West Palm PBCH). Trimble GPS Pathfinder Office was used to create data dictionaries and to export the spatial data into ESRI shapefile format. ArcGIS version 10.4.1 was used to edit shapefiles and to create maps. We used the Universal Transverse Mercator (UTM) projection (zone 17 N) as our coordinate system for all spatial data (datum = WGS 1984).

**Figure 2.**
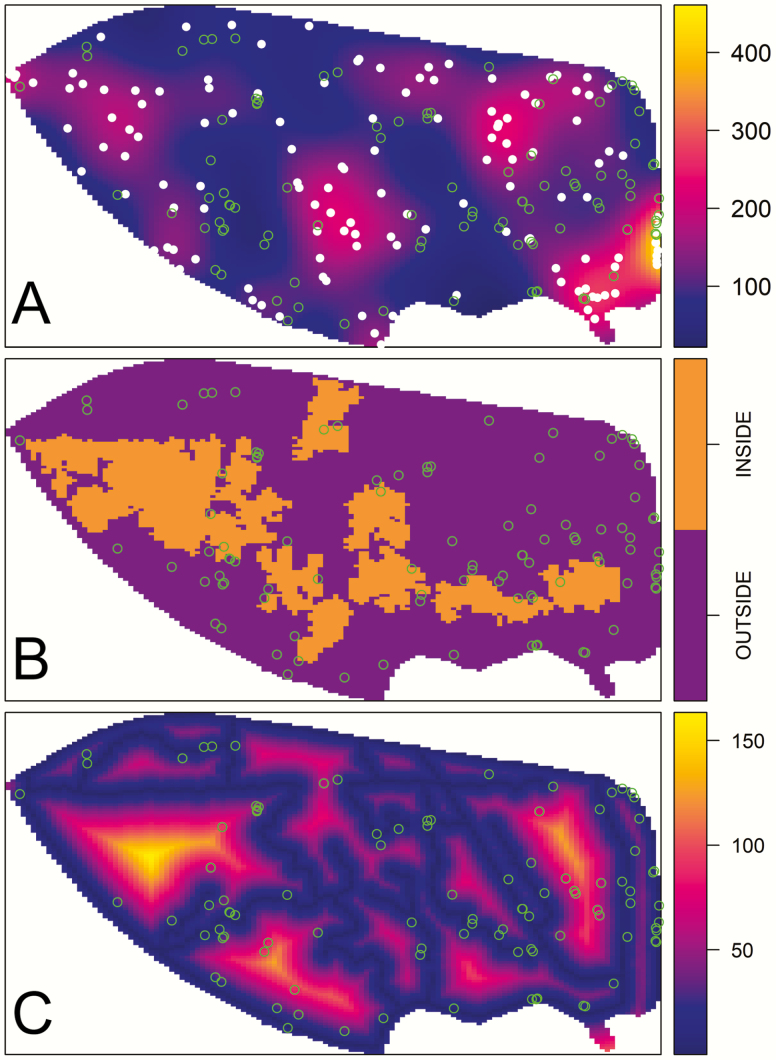
Covariate surfaces used as predictors of *Chrysobalanus icaco* density in inhomogeneous Poisson point process models: (A) kernel-smoothed density surface (in points per kilometre) for *Gopherus polyphemus* burrows (white dots), (B) areas INSIDE (= orange) and OUTSIDE (= purple) of *Serenoa repens* patches and (C) distance (in meters) from *G. polyphemus* movement trails (for the analysis distance was rescaled to kilometres). The locations of *C. icaco* bushes (green dots) are superimposed on each surface. The bounding box surrounding each surface is 1.65 × 0.86 km.

**Figure 3.**
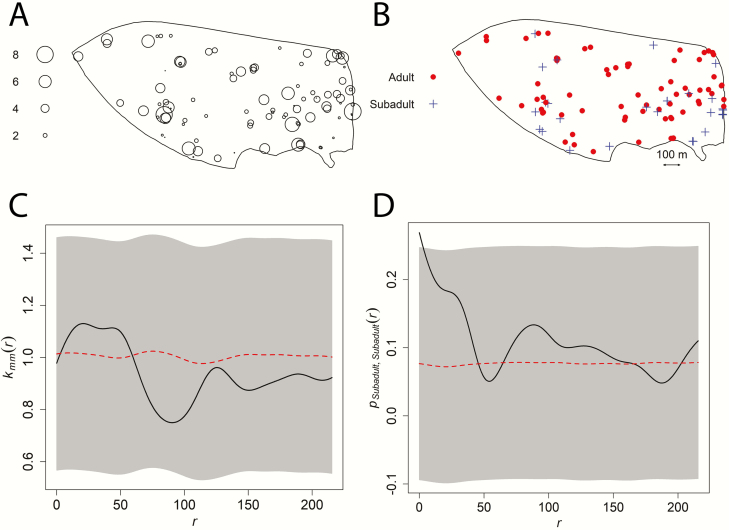
(A) Map showing the relative height of *Chrysobalanus icaco* within the study area, where circle size is proportional to height (in meters). (B) *Chrysobalanus icaco* height was binned into two categories representing adult bushes (≥1.5 m; red dots) and subadult/seedling bushes (˂1.5 m; blue plus symbols). The mark correlation Function (C) was based on height of *C. icaco* bushes (shown in panel A); the mark connection Function (D) was based on the age/class categories (shown in panel B). The significance of the mark correlation and mark connection functions were determined by random labelling (number of randomizations = 39; the minimum number of randomizations required for a two-sided test at a significance level of 5 %). The black lines in each figure represent the mark correlation functions and mark connection functions (respectively). The red dashed lines show the reference values that would be expected under the null hypothesis of random labelling. The grey bands represent the global significance envelopes under the null hypothesis of random labelling (for the Monte Carlo tests). Note that the mark connection function wanders outside the significance envelope, leading to the rejection of the null hypothesis of random labelling.

Coordinates for *C. icaco* bushes (*n* = 99; Figs 1 and 2) were collected during the fruiting season. A tape measure was used to determine the height of each plant (Fig. 3A). Informal observations of *C. icaco* bushes in the study area indicates that most bushes reached adult size in ~5 years, at a height ranging from 1 to 2 m. A *C. icaco* bush was classified as an adult if it had reached adult size and was observed fruiting or flowering during the survey; all other bushes were combined into a single category that contained both seedling and subadult bushes (Fig. 3B). Tortoise burrows (*n* = 132; Fig. 2A) were targeted to obtain coordinates based on archival maps of the site made during previous field observations. New burrows encountered during additional site surveys were also mapped. We considered all known burrow locations, regardless of their activity status. The study area boundary (Fig. 1) was mapped by travelling the perimeter with the GNSS receiver. Lower elevation habitats were excluded because it is possible that past flood events in the catchment basin may have allowed for seed movement not attributable to tortoise activity. The GNSS receiver was also used to map the boundaries of *S. repens* thickets (*n* = 10; Fig. 2B). *Serenoa repens* thickets were mapped as polygons by tracing their perimeter as closely as possible. The *S. repens* thickets may act as exclusion zones for both tortoise movement and *C. icaco* seed germination because the recumbent trunks often made walls of vegetation. *Serenoa repens* thickets were spared during the last intensive reduction mowing operation in 2006 if they encircled known burrows at the time.

As the study area became overgrown in the decade since the reduction mowing was performed, tortoises have maintained pathways used to travel from areas of grazing to their burrow sites, as well as pathways to connect to high traffic areas and highly frequented female burrows. Those trails are identifiable in many ways: by archival data showing pathways created by motocross bikes which can still be seen in satellite images, by identifying passages made through thick understories and maintained by constant animal traffic and by personal observation of tortoises traversing pathways while roaming for grazing or mating. The *G. polyphemus* trails were often connected to the two recreational paths maintained by county mowers, as well as to the running paths that encircle the study boundary, and provide for basking and foraging areas adjacent to the wooded understory. *Gopherus polyphemus* movement trails (*n* = 20) were walked with the GNSS receiver and mapped as a polyline file (Fig. 1A).

### Analyses

#### Germination analysis.

To test whether counts of germinated seeds were proportional across treatments, we used the chi-square test of homogeneity (α = 0.05). We plotted germination curves as the cumulative relative frequency of seeds that had germinated by each day of the germination study. To compare germination patterns among treatment groups, we used time to event analysis (McNair *et al.* 2012), which is based on comparison of survival functions. In the context of germination analysis, a seed survival function is calculated as one minus the cumulative relative frequency of seeds germinated by each time step, and it is interpreted as the proportion of seeds not yet germinated (or as the probability that germination occurred after a certain day in the experiment). We chose the non-parametric version of time to event analysis based on the Kaplan–Meier survival estimator (Kaplein and Meier 1958) because it can account for right censored cases, here defined as seeds that did not germinate by the end of the observation period. We used both log-rank (Mantel–Cox) and Breslow (generalized Wilcoxon) tests (α = 0.05) to determine if differences in germination curves existed across the three treatments, where the latter test (Breslow) is more sensitive to early events. Breslow tests were also used to conduct *post hoc*, pairwise comparisons between treatment groups with Bonferroni correction (α = 0.05/3 = 0.017). All statistical analyses related to the germination study were conducted using SPSS version 24 (IBM Corp 2016).

#### Point pattern analysis.

We used point pattern analysis to characterize the spatial distribution of *C. icaco* bushes in our study area and to determine if variation in the spatial density (an estimate of the intensity of the underlying point process) of *C. icaco* could be explained by dependency on particular environmental covariates. In point pattern analysis, it is common practice to start with a simple null model of a random point process, typically a homogeneous Poisson point process, also known as complete spatial randomness (CSR). Homogeneity pertains to the assumption that the average intensity of the point process is constant in space, such that the counts of points in different subregions represent random samples from a distribution with the same mean intensity. If the null model of a homogeneous Poisson point process is rejected, then other types of point process models should be evaluated. An inhomogeneous Poisson point process is a modification of CSR in which the average intensity of points varies, due to dependency of the intensity function on one or more underlying environmental covariates. If an inference of clustering between points remains after correction for inhomogeneity, then some type of cluster process may be more appropriate.

We used likelihood-ratio tests (α = 0.05) to compare inhomogeneous Poisson point process models containing one or more spatial covariates to nested subsets, where the null (intercept only) model was a homogeneous Poisson point process (i.e. no effect of a change in the value of a covariate on the log density of points). We examined how the log density of *C. icaco* bushes within the study area was affected by: (i) a change in the density of *G. polyphemus* burrows, (ii) the distance to *G. polyphemus* movement pathways, (iii) whether *C. icaco* bushes were situated inside or outside of *S. repens* patches and (iv) the coordinates *x* and *y* (which were used to examine the change in *C. icaco* density relative to the direction of the four founder plants.

To estimate the density of *G. polyphemus* burrows at different positions within the study area, we used kernel smoothing (Fig. 2A). The bandwidth of the smoothing kernel was chosen via maximum likelihood cross-validation (Diggle 1985). To create a logical covariate surface representing whether *C. icaco* bushes were located inside or outside of *S. repens* patches, we converted polygon shapefiles into a factor-valued function (Fig. 2B). To estimate the distance from all positions in the study area to gopher tortoise movement pathways, we created a distance function from the line segment patterns (Fig. 2C).

To determine whether *C. icaco* bushes were more clustered than expected after accounting for dependency on environmental covariates, we conducted a one-sided global envelope test (alternative = ‘greater’; α = 0.05) based on the inhomogeneous version of Besag’s *L* function (a variance-stabilized transformation of Ripley’s *K* function with correction for inhomogeneity). The observed inhomogeneous *L* function was compared to the range of expected *L* functions based on multiple simulations (= 19; the minimum number of simulations need for a one-sided test at a significance level of 5 %) from a fitted inhomogeneous Poisson point process model, containing covariates that were supported by the likelihood-ratio tests. To account for conservatism associated with the composite null hypothesis, we fixed the number of simulated points in each simulation to equal to the number of *C. icaco* bushes (= 99). To examine the effect of correction for inhomogeneity on the *L* function, results for the inhomogeneous *L* function were compared with the results of a one-sided global envelope test for the standard (i.e. uncorrected) version of Besag’s *L* function. Because the study area was sampled completely to its boundaries for *C. icaco*, we did not use edge correction when calculating empirical *L* functions.

If *C. icaco* dispersal were restricted, then shorter *C. icaco* bushes would tend to be near parental bushes. Alternatively, if there were clump dispersal of seeds in faeces, then seeds of similar height or age class should tend to be closer together. We used mark connection analysis to examine whether age class categories (i.e. adult versus subadult/seedling) were more similar or dissimilar than expected at varying distances between bushes. For the numeric variable plant height, we used mark correlation analysis to test whether plant height was more similar or dissimilar than expected at varying distances between bushes. The significance of the mark correlation and connection coefficients was determined by comparing the coefficient values for the observed pattern to the range of expected values for each coefficient when the labels (i.e. heights or age classes) were randomized among the *C. icaco* locations (number of randomizations = 39; the minimum number of randomizations required for a two-sided test at a significance level of 5 %).

All point pattern analyses were conducted with the spatstat package (Baddeley *et al.* 2015) in R version 4.0.0 (R Core Team 2020). We also used functions from the maptools and rgdal packages in R version 4.0.0 to convert ESRI shapefiles into spatial objects that could be interpreted by spatstat.

## Results

### Germination experiment

**Figure 4.**
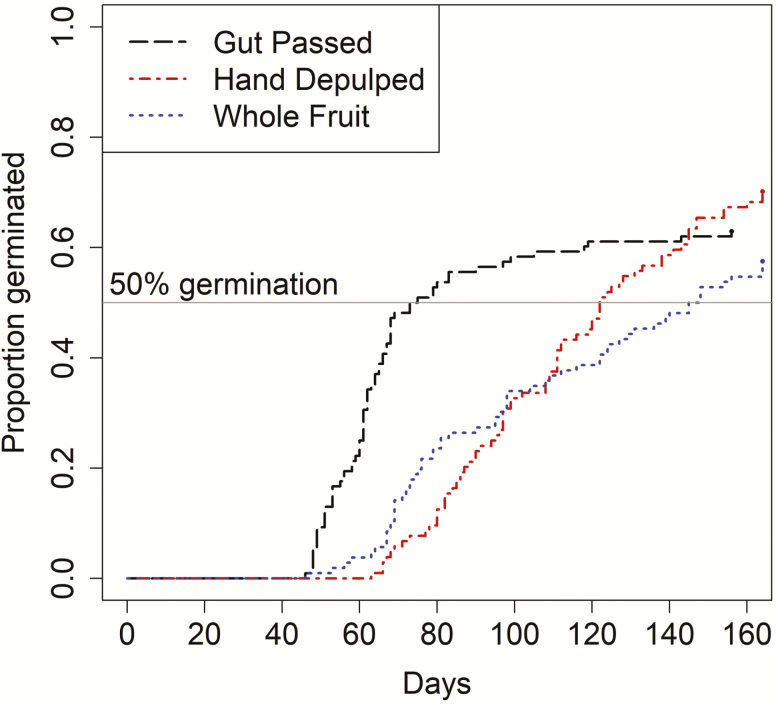
Germination curves for the three treatments from the germination experiment: gut passed (black long dashed line), hand depulped (red dot and dashed line) and whole fruit (blue dotted line). Germination curves were plotted as the cumulative relative frequency of seeds that had germinated by each observation day over the 165-day period. In total, 108 gut-passed seeds, 104 hand-depulped seeds and 106 whole fruits were used in the experiment; 68 of 108 gut-passed (= 63 %), 73 of 104 hand-depulped (= 70.2 %) and 61 of 106 whole fruit (= 57.5 %) seeds germinated by the end of the experiment. The horizontal grey line represents the threshold for 50 % germination.

In total, 68 of 108 gut-passed (= 63 %), 73 of 104 hand-depulped (= 70.2 %) and 61 of 106 whole fruit (= 57.5 %) seeds germinated during the time frame of the experiment. The total proportion of seeds that germinated did not differ significantly among treatment groups (χ ^2^ = 3.645, df = 2, *P* = 0.162). The hand-depulped and whole fruit treatments took considerably longer than gut-passed seeds to reach 50 % germination (Fig. 4): 95 and 108 days for hand-depulped and whole fruit treatments, respectively, compared with only 61 days to 50 % germination for gut-passed seeds. Seeds within the gut-passed treatment had a median time to germination of 73 days (95 % CI: 61.4–84.7 days), which was less than the median time to germination for the hand-depulped treatment (median = 122 days; 95 % CI: 109.5–134.5 days) and the whole fruit treatment (median = 145 days; 95 % CI: 119.8–170.2 days).

Both statistical tests from the time to event analysis supported significant differences in germination curves (Fig. 4; log-rank test: χ ^2^ = 6.794, df = 2, *P* = 0.033; Breslow test: χ ^2^ = 21.709, df = 2, *P* < 0.001). *Post hoc* pairwise tests identified significant differences in germination curves between the gut-passed and hand-depulped treatments (χ ^2^ = 13.757, df = 1, *P* < 0.001) and between the gut-passed and whole fruit treatments (χ ^2^ = 14.836, df = 1, *P* < 0.001), but no significant differences were detected between the hand-depulped and whole fruit treatments (χ ^2^ = 0.535, df = 1, *P =* 0.464).

### Point pattern analysis

For *C. icaco* bushes, there was strong evidence against a null model of a homogeneous Poisson point process, in favour of an inhomogeneous Poisson point process that included any of the examined covariates, except the kernel-smoothed density of *G. polyphemus* burrows and the *y*-coordinate. While we did not initially detect a significant effect of burrow density on *C. icaco* density, regression diagnostics revealed several sets of highly influential *C. icaco* bushes located at the extreme east (*n* = 8) and west edges (*n* = 1) of the study area **[see****Supporting Information—Fig. S2****]**, in subregions with very high burrow density (Fig. 2A). When these data points were removed from the analysis, there was strong evidence against a homogeneous Poisson point process in favour of a model that included the burrow density surface as a covariate (χ ^2^ = 9.34, df = 1, *P* = 0.002). For the model that was best supported by the data (Tables 1 and 2) regression coefficients (β) for the different predictors indicated a statistically significant decrease in the log density of *C. icaco* with an increase in *G. polyphemus* burrow density (β = −0.0095, SE = 0.0025, *P* < 0.001), an increase in the log density of *C. icaco* from west to east (β *=* 0.98, SE = 0.28, *P* < 0.001), a decrease in the log density of *C. icaco* with increasing distance from *G. polyphemus* movement paths (β = −11.72, SE = 4.84, *P* = 0.008) and a decrease in the log density of *C. icaco* inside (as opposed to outside) *S. repens* patches (β = −1.19, SE = 0.43, *P* = 0.003). A model including third-degree terms for both the *x*-coordinate and distance from trails further improved model fit, with the exception of a few small subregions in the study area where *C. icaco* density was persistently over or underestimated based on the model **[see****Supporting Information—Fig. S3****]**.

**Table 1.** Results of likelihood-ratio tests of the null hypothesis of a homogeneous Poisson point process (= intercept-only model) against the alternative of an inhomogeneous Poisson process with intensity that is a log-linear function of one or more predictors, represented as nested subsets. The response variable was *Chrysobalanus icaco* density; the fully specified model contained four predictors (plus the intercept term): the kernel-smoothed density of *Gopherus polyphemus* burrows (= Burrow density), the *x*-coordinate (= *x*), the distance to the nearest movement pathway (= Trail distance) and whether or not a point was located inside or outside of a *Serenoa repens* thicket (= *Serenoa repens*). Deviance: change in deviance between model and nested model, which is chi square distributed with the specified number of degrees of freedom (= df). ~ indicates predictors in the log-linear models. Results support a model containing all four predictor variables over nested subsets.

Model	df	Deviance	*P-*value
~ 1 (intercept only)			
~ Burrow density	1	9.34	0.002
~ Burrow density + *x*	1	17.39	< 0.001
~ Burrow intensity + *x* + Trail distance	1	11.55	= 0.001
~ Burrow intensity + *x* + Trail distance + *Serenoa ripens*	1	10.60	= 0.001

**Table 2.** Regression coefficients (β), standard errors of beta (SE), *Z*-score based on Wald test (*Z*) and corresponding *P-*values (two-tailed) for the inhomogeneous Poisson point process model that was best supported by likelihood-ratio tests (Table 1). The response variable was the log density of *Chrysobalanus icaco* (bushes per km^2^); significant predictors of *C. icaco* density included: (i) the kernel-smoothed density of *Gopherus polyphemus* burrows (burrows per km^2^), (ii) the *x*-coordinate (in meters), (iii) the distance (in kilometres) from *G. polyphemus* movement trails and (iv) a factor-valued function indicating whether *C. icaco* bushes were located inside or outside *Serenoa ripens* thickets (reference level was OUTSIDE thicket).

Site	β	SE	*Z*	*P-*value
Intercept	−919.60	264.24	−3.48	= 0.001
Burrow density	−0.0095	0.0025	−3.86	= 0.001
*x*	0.98	0.28	3.50	< 0.001
Trail distance	−11.72	4.83	−2.42	0.015
INSIDE	−1.19	0.43	−2.77	0.006

**Figure 5.**
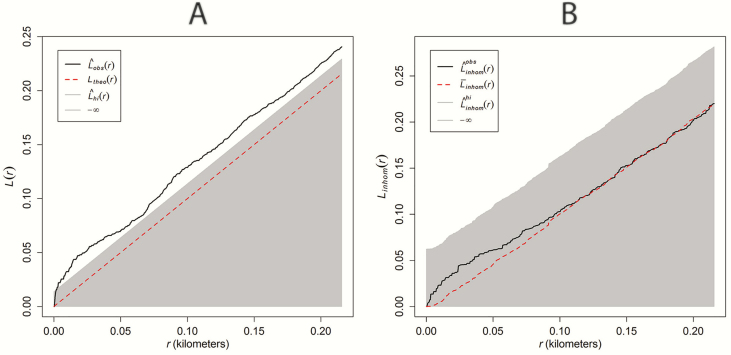
(A) *L* function and (B) inhomogeneous *L* function for *Chrysobalanus icaco* bushes within the 9.27-ha study area in Jupiter, FL, USA. The solid black line represents the observed *L* function (A) and observed inhomogeneous *L* function (B) over increasing distance (*r*) in kilometres. The grey area depicts the 5 % simultaneous significance envelope and the stippled red line represents the average *L* function (A) or inhomogeneous *L* function (B) for simulated locations. Note that the observed *L* function (A) wanders far above the 5 % significance envelope, indicating strong evidence against the null hypothesis of a homogeneous Poisson point process in the direction of clustering. After correcting for inhomogeneity (B), there was still some tendency towards clustering (black line deviates above stippled red line), but the observed function was well within the range of what might be observed under the null hypothesis of an inhomogeneous Poisson point process. These results suggest virtual aggregations of *C. icaco* associated with dependency on environmental covariates, rather than clustering associated with an endogenous spatial process.

After correcting the *L* function for inhomogeneity, there was some weak but non-significant clustering of bushes at short distances (Fig. 5). Mark correlation analysis (Fig. 3C) also revealed weak positive autocorrelation of plant height at short distances between bushes, but the magnitude of the correlation coefficient was within the range of what might be observed under the null hypothesis of random labelling of height. For the mark connection analysis (Fig. 3D), the null hypothesis of random labelling was rejected due to greater than expected contiguity of subadult/seedling bushes at very short distances (<10 m).

## Discussion

### Germination of Cocoplum seeds

Our germination experiment revealed that *C. icaco* seeds that were found in *G. polyphemus* faeces germinated earlier than seeds that were hand–depulped or planted as whole fruits, meaning that there was only a scarification effect. Although we found differences in the timing of germination, by the end of the experiment, we did not detect a significant difference in the total proportion of seeds that had germinated in each group.

Scarification of the seed coat has been purported to aid germination in some plants, as the tissues surrounding the embryo could impede the radicle and/or hypocotyl from protruding and signalling germination success (Traveset 1998; Traveset and Verdú 2002; Traveset *et al.* 2007). In prior germination trials involving *C. icaco*, Daley and Zimmerman (2007) reported that manual and acid scarification did not affect the total proportion of seeds germinated or the speed of germination. Francis and Rodriguez (1993) reported a high germination percentage for *C. icaco* seeds (89 %) starting at 34 days after planting in potting soil, with scarification of seeds not being required (Francis 2004). In contrast, our study indicates that scarification associated with gut passage through *G. polyphemus* resulted in earlier germination of *C. icaco*.

For *C. icaco*, the whole fruit treatment was designed to simulate how seeds would fare if fruit dropped to soil and became partly desiccated before germination (Samuels and Levey 2005; Robertson *et al.* 2006). Although there were some visible differences in germination patterns for the hand-depulped versus whole fruit treatments (Fig. 4), the germination curves were not significantly different, supporting the idea that deinhibition did not account for differences in the timing of germination observed for gut-passed seeds.

Early germination provides a competitive growth advantage that has been well-documented in many plant species (Black and Wilkinson 1963; Ross and Harper 1972; Jones *et al.* 1997; Seiwa 1998; Orrock and Christopher 2010), and may be especially important in plant invasions (Gioria *et al.* 2018). However, if and how early germination may be advantageous for *C. icaco* seedlings is difficult to ascertain without additional field experiments. Nevertheless, certain benefits that have been associated with early germination in other systems, such as light availability in deciduous forests (Seiwa 1998), seem unlikely to be advantageous for *C. icaco* in our study area where the growing season is long and most canopy trees are evergreen or semi-evergreen; instead, other benefits of early germination, such as higher seedling survival associated with lower susceptibility to attack from predators and pathogens (Seiwa 1998; Abe *et al.* 2008), may be more plausible. Early germination of *C. icaco* seeds could also provide a competitive advantage over other plants species whose seeds may co-occur in faeces with *C. icaco* seeds. During faecal dissections, a number of grass seeds and other small seeds were also observed in *G. polyphemus* faeces, but were not identified. The only seeds observed that were similar in size to *C. icaco* were from *S. repens* and *L. michauxii*. However, overall abundances of *S. repens* fruits and seeds were relatively low within our study area due to illegal collection of fruits for the herbal medicine trade, and only one faecal sample contained a seed from *L. michauxii* (and did not contain any seeds from *C. icaco*). It is therefore conceivable that faster germination of *C. icaco* associated with gut passage could result in competitive advantages in terms of growth over seeds of other plants species that may be ingested by *G. polyphemus*, but such interspecific competition among plants with similar seed dispersal syndromes did not appear to be severe within our study area.

Seed dormancy is a potential confounding factor that could have affected our germination trial results if date of collection affected whether or not a seed was dormant (Bewley 1997; Finch-Savage and Leubner-Metzger 2006). However, date of collection did not seem to have noticeable effect on the timing of germination in our trials. For example, two gut-passed seeds were collected 63 days apart but germinated simultaneously on Day 53. Likewise, two other gut-passed seeds were collected 82 days apart and both germinated on Day 68.

Studies of other tortoise species have found variable effects of ingestion on seed germination. Rick and Bowman (1961) found that gut passage through *Chelonoidis porteri* (Galápagos Tortoise) enhanced both the speed and total percentage of seeds germinated in the *Lycopersicon esculentum* (Galápagos Tomato). However, a more recent study of *C. porteri* by Blake *et al.* (2012) found that gut passage did not increase germination success (i.e. defined as whether or not a seed germinated) in five plant species that were commonly found in dung piles, but that long gut retention times and movement patterns promoted long-distance dispersal of seeds. Analogous studies of *Testudo graeca* (Spur-Thighed Tortoise; Cobo and Andreu 1988), *Chelonoides chilensis* (Chaco Tortoise; Varela and Bucher 2002) and *Chelonoides denticulata* (Amazonian Yellow-Footed Tortoise; Guzmán and Stevenson 2008; Jerozolimski *et al.* 2009) reported varying effects of ingestion on seed germination depending on the plant species consumed. Most of the studies listed above reported high diversity of ingested seed species, relatively long gut retention times and the potential for tortoise species to be effective long-distance seed dispersers.

Previous studies of endozoochoric dispersal by tortoises suggest that tortoises may be particularly important dispersal agents for large-seeded plant species. For example, Guzmán and Stevenson (2008) reported that one (*Rauvolfia micrantha*: Small-flowered Snakeroot) of the two plant species that exhibited a higher percentage of germination after gut passage through *C. denticulata* was large-seeded, and Jerozolimski *et al.* (2009) found that *C. denticulata* may be only one of a few dispersal agents capable of ingesting several large-seeded species (such as *Attalea maripa* (Maripa Palm) and *Spondias mombin* (Yellow Mombin)). Griffiths *et al.* (2011) found that introduced *Aldabrachelys gigantea* (Aldabra Giant Tortoises) enhanced the speed and percentage of germination for the large-seeded fruits of the endangered *Diospyros egrettarum* (Ebony Tree), and the introduction of the Aldabra Giant Tortoise to Ile aux Aigrittes (near Mauritius) restored a dispersal mutualism that was lost with the extinction of the endemic *Cylindraspis* tortoises. Several recent studies have also highlighted the potential for tortoises to facilitate germination and dispersal of exotic fleshy fruited plants with large seeds, such as *Mimusops coriacea* (Monkey’s Apple; Waibel *et al.* 2013). Although long gut retention times in tortoises have generally been thought to facilitate dispersal and germination of large-seeded fruits, there may be diminishing returns with increasing body size in giant tortoises due to prolonged passage times that may increase the probability of seeds being damaged or destroyed (Waibel *et al.* 2013; Falcón *et al.* 2020).

### Spatial distribution of Cocoplum

The results of our point pattern analysis support the hypothesis that *G. polyphemus* facilitated the rapid westward dispersal of *C. icaco* within our study area by dispersing *C. icaco* seeds along the surface of linear movement pathways. The fact that relatively tall (presumably older) *C. icaco* bushes were spread across the study area (Fig. 3A) indicates that dispersal of *C. icaco* occurred rapidly and was likely followed by subsequent waves of dispersal from new plants that established and reached sexual maturity.

Following ingestion, *G. polyphemus* presumably deposit seeds randomly in areas where they are active, including along moving trails and within burrows (Young and Goff 1939; Hansen 1963; Ultsch and Anderson 1986). The negative correspondence between areas of intensive burrowing and *C. icaco* recruitment is most likely explained by the assumption that seeds that are defecated deep within burrows are unlikely to germinate and establish without secondary movement. While *C. icaco* density was generally lower in areas with many burrows, our point pattern data support the observation that some *C. icaco* germinated on the apron of *G. polyphemus* burrows, most likely due to faecal deposition of seeds near the entrance or by ejection of deeply deposited seeds when burrows were cleaned out by tortoises.

After accounting for inhomogeneity, statistically significant clustering of *C. icaco* was not supported (Fig. 5). This indicates that most aggregations of *C. icaco* in our study were due to dependency on spatial covariates that affect the numbers of *C. icaco*, rather than due to an endogenous cluster process (Li *et al.* 2016). Lack of evidence supporting a cluster process for *C. icaco* is consistent with the observation that most faeces contained only one to a few *C. icaco* seeds. Occasional clump dispersal of seeds in faeces could explain higher than expected proximity of young plants at short distances, as revealed by the mark connection analysis (Fig. 3D). Overall, however, the results of our point pattern analysis indicate that if clusters do exist, they are relatively small, on average.

Linking seed dispersal activity with adult vegetation structure is a notoriously challenging problem in plant ecology due to the large number of potential confounding factors (Schupp and Fuentes 1995; Nathan and Muller-Landau 2000; Wang and Smith 2002), such as intricate networks of primary and secondary dispersal agents and seedling mortality. While we cannot rule out diffuse interactions with other seed dispersers, the large size of *C. icaco* fruit and seed precludes movement by many common frugivores, such as songbirds and small mammals (Wheelwright 1985; Bartlow *et al.* 2011). The birds most capable of dispersal at this site are *Dryocopus pileatus* (Pileated Woodpecker) and *Corvus ossifragus* (Fish Crow), but both were rarely observed at the study site. *Chrysobalanus icaco* could potentially be dispersed by Canids (namely *Vulpes vulpes* (Red Fox), *Urocyon cinereoargenteus* (Gray Fox) and *Canis latrans* (Coyote)), but there was little evidence of Canid activity within our study area; only transient individuals and small groups moving through the area were observed, and *C. icaco* seeds have never been found in their faeces within the study area. *Procyon lotor* (Raccoon) could have conceivably dispersed *C. icaco* seeds, but *P. lotor* population density was low during most of the study. When *P. lotor* droppings were encountered, none contained *C. icaco* seeds, even at the peak of *C. icaco* fruiting. Although it is possible that some *C. icaco* seeds were dispersed by other species beyond *G. polyphemus*, the preponderance of *C. icaco* seeds in *G. polyphemus* droppings, and the lack of evidence supporting other seed dispersers, implicates *G. polyphemus* as the main seed disperser within our study area.

At other locations, *G. polyphemus* has been identified as a seed dispersal agent for other native drupe plants. In scrub habitats, *Ximenia americana* (Hog Plum) seeds have been found in burrow aprons, and individual plants are frequently found sprouting next to *G. polyphemus* burrows at Florida Atlantic University Harbor Branch Oceanographic Institute campus in Fort Pierce, FL, USA (J. A. Moore, pers. obs.). In both scrub and flatwoods, *L. michauxii* fruits are a well-known food item for *G. polyphemus* (Austin 1990; Ashton and Ashton 2008) and adult plants are often near tortoise burrows or commonly used foraging areas (J. A. Moore, pers. obs.). Seeds of both *X. americana* and *L. michauxii* have been recorded in the faeces of *G. polyphemus* at other locations, such as at the Archbold Biological Station in Venus, FL, USA (Carlson *et al.* 2003), and at least one *L. michauxii* seed was noted in a faeces at our study site (J. A. Moore, pers. obs.).

Although there have been numerous accounts of endozoochoric dispersal of large-seeded plants by *G. polyphemus*, our study is the first to demonstrate a non-random correspondence between the recruitment patterns of a large-seeded plant species and *G. polyphemus* activity patterns. Over the last decade, point pattern analysis has been used increasingly in the seed ecology literature to examine the correspondence between disperser behaviour and plant recruitment patterns (Wiegand *et al.* 2009; Fedriani *et al.* 2010; Puerto-Piñero *et al.* 2010; Jara-Guerrero *et al.* 2015; Valenta *et al.* 2015). However, our study is the first to apply these methods to study the relationship between a plant and a reptilian dispersal agent.

### Conclusions and future directions

While *C. icaco* is known to disperse long distances by water, our results demonstrate that colonization of upland areas may be dependent on animal dispersal agents that are attracted to its fruits and capable of swallowing its seeds and fruit. In addition to moving seeds away from parental plants, endozoochory of *C. icaco* by *G. polyphemus* scarified the seed coat in a manner that resulted in earlier germination than was observed for seeds that were hand-depulped or seeds contained within whole, dried fruits, meaning there was only a scarification, and not a deinhibition effect on germination speed. Further work is required to determine if early germination provides a competitive advantage in a field setting for *C. icaco*.

Our results support the potential for the rapid colonization of linear networks (here: natural and anthropogenic trail systems) with fleshy fruited shrubs by seed deposition associated with animal dispersers (Suárez-Esteban *et al.* 2013a,b; Suárez-Esteban *et al.* 2016). Studies that track the fate of *C. icaco* seeds that fall underneath parental plants and in different microhabitat settings could provide further insight into the conditions favouring *C. icaco* germination and establishment. Likewise, studies that compare germination and establishment of *C. icaco* from faeces bearing varying numbers of seeds could provide further details about the distance and/or density-dependent mechanisms that affect *C. icaco* recruitment (Janzen 1970, 1971; Connell 1971; Clark and Clark 1984).

Our results also highlight the potential for the rapid dispersal and/or naturalization of ornamental varieties of *C. icaco* if it is introduced to areas containing a large population of *G. polyphemus*. Since our study was based on just one site and set of founder plants, additional studies that use similar methods of spatial analysis at other sites where *C. icaco* and *G. polyphemus* are syntopic would be helpful in determining if our results can be applied to other areas. While further studies are required, our findings serve as a warning concerning the use of introduced tortoises in land management and conservation efforts. If an invasive plant with seeds that can pass through tortoises occupies such land conservation reserves, then tortoises could inadvertently become the dispersers of those invaders, potentially leading to dramatic and rapid changes in community composition and ecosystem function.

## Supporting Information

The following additional information is available in the online version of this article—

**Figure S1.** Frequency distribution of *Chrysobalanus icaco* seed counts from the 34 *Gopherus polyphemus* scats recovered from the study area within Abacoa Greenway in Jupiter, FL, USA. The number of *C. icaco* seeds per scat ranged from 1 to 20 (median = 3, mode = 1).

**Figure S2.** (A) Influence of each data point (*n* = 99; sorted in ascending order of influence) on the inhomogeneous Poisson point process model with *Chrysobalanus icaco* density as the response variable and the kernel-smoothed density of *Gopherus polyphemus* burrows as the predictor. The plot shows that there were eight data points with relatively high influence measures (red dots). (B) Map showing the locations of *C. icaco* bushes, with influence represented by the relative size of the circle; the circles in red correspond to the eight influential data points (from panel A) that had an influence measure greater than or equal to 0.03. The map shows that *C. icaco* bushes on the extreme east edge of the study area had a disproportionally strong impact on the results of the log-linear model. When these points were removed from the analysis, the kernel-smoothed density of *G. polyphemus* burrows became a significant predictor of *C. icaco* density.

**Figure S3.** (A) Smoothed Pearson residual field for the inhomogeneous Poisson point process model containing third-degree terms for both the *x*-coordinate and distance from trails (see text for details). The ribbon on the right shows the corresponding colour map for the values of the smoothed Pearson residuals. (B) Logical function showing areas where the absolute value of the smoothed Pearson residual field exceeded two standard deviations (= TRUE, in orange). Locations of *Chrysobalanus icaco* bushes (green dots) are superimposed on each surface. Overall, the model performed well, except for several subregions where counts were significantly higher or lower than predicted.

plaa024_suppl_Supplementary_Information

## Data Availability

The data used in this article are available from: https://data.mendeley.com/datasets/twvc6d3dg4/1

## Sources of Funding

The Harriet L. Wilkes Honors College-Florida Atlantic University provided funding to support the germination trials and application processing charges. Application processing charges were also partly funded by the College of Science and Mathematics-Valdosta State University.

## Contributions by the Authors

This manuscript is based on the MS thesis of C.J.H., conducted at Florida Atlantic University under the supervision of J.A.M. C.J.H. and J.A.M. conceived of the idea for the project. C.J.H., S.V. and J.A.M. developed methodology and conducted field work. C.J.H. and S.V. maintained germination trials; designed and analysed by C.J.H. Point pattern analyses were conducted by C.D.A. C.J.H. and C.D.A. wrote the manuscript, with input from J.A.M. and S.V.

## Conflict of Interest

None declared.
