# Supplementary material for: Endozoochory of Chrysobalanus icaco (Cocoplum) by Gopherus polyphemus (Gopher Tortoise) facilitates rapid germination and colonization in a suburban nature preserve
Source: AoB Plants. 2020 Jun 19;12(4):plaa024. doi: 10.1093/aobpla/plaa024 (PMC7363059; doi:10.1093/aobpla/plaa024)
Supplement: plaa024_suppl_Supplementary_Information [file plaa024_suppl_supplementary_information.docx]

**
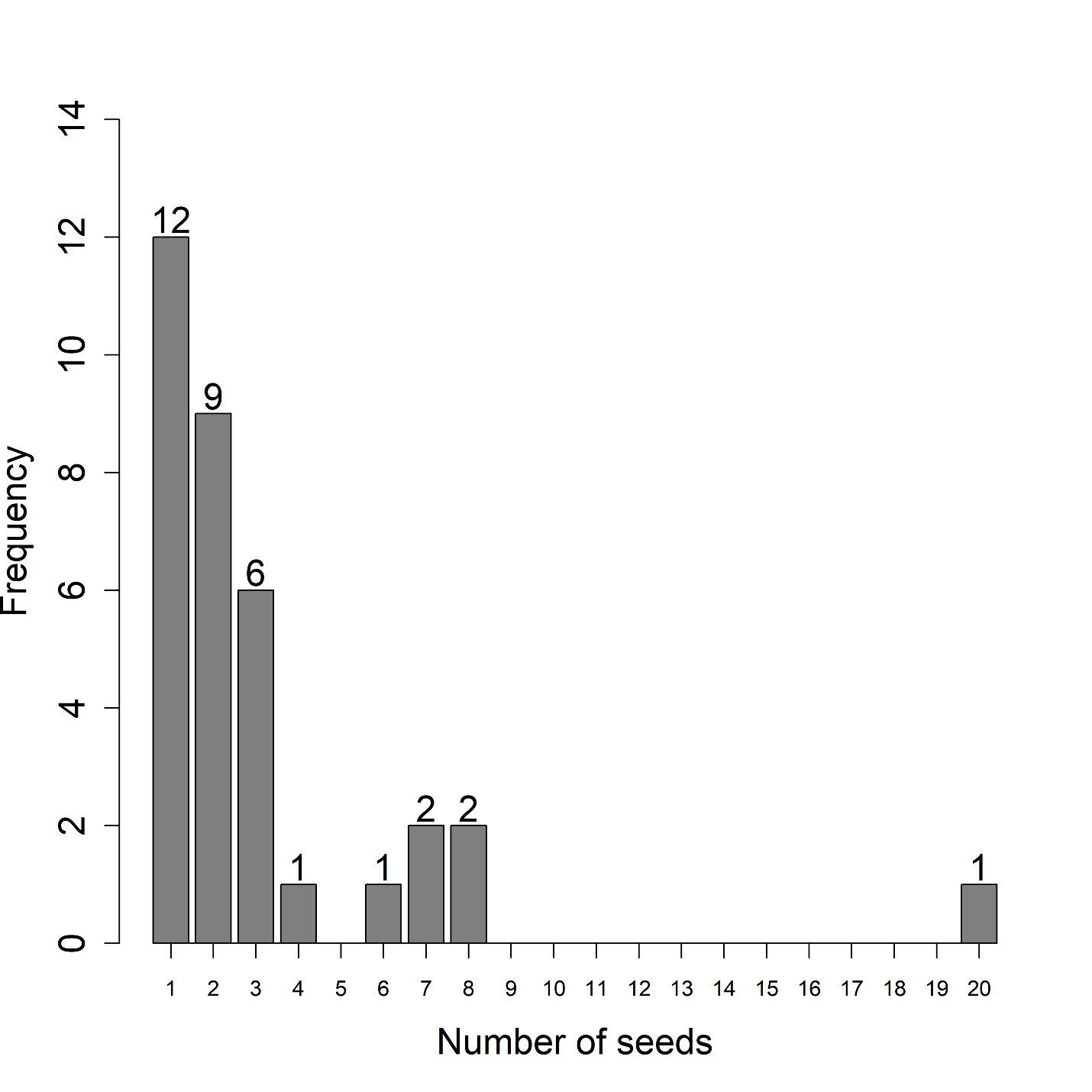
**

**Figure S1.** Frequency distribution of *Chrysobalanus icaco* seed counts from the 34 *Gopherus polyphemus* scats recovered from the study area within Abacoa Greenway in Jupiter, Florida, USA. The number of cocoplum seeds per scat ranged from 1 to 20 (median = 3, mode = 1).

**
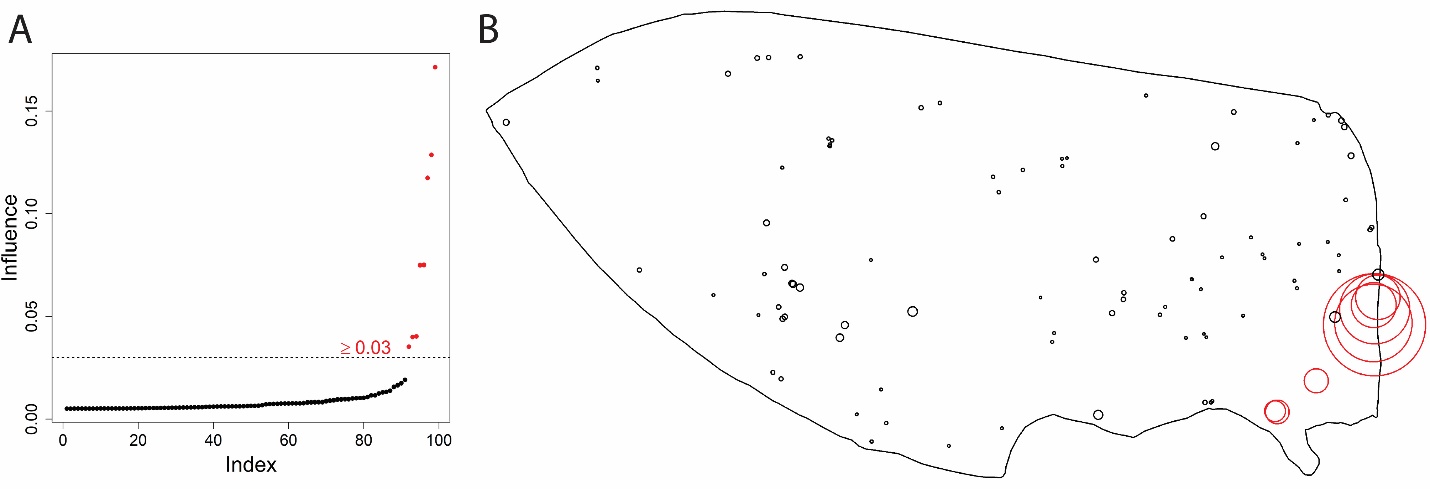
**

**Figure S2.** (A) Influence of each data point (n = 99; sorted in ascending order of influence) on the inhomogeneous Poisson point process model with *C. icaco* density as the response variable and the kernel smoothed density of gopher tortoise burrows as the predictor. The plot shows that there were eight data points with relatively high influence measures (red dots). (B) Map showing the locations of *C. icaco* bushes, with influence represented by the relative size of the circle; the circles in red correspond to the eight influential data points (from panel A) that had an influence measure greater than or equal to 0.03. The map shows that *C. icaco* bushes on the extreme east edge of the study area had a disproportionally strong impact on the results of the log-linear model. When these points were removed from the analysis, the kernel smoothed density of gopher tortoise burrow became a significant predictor of *C. icaco* density.

**
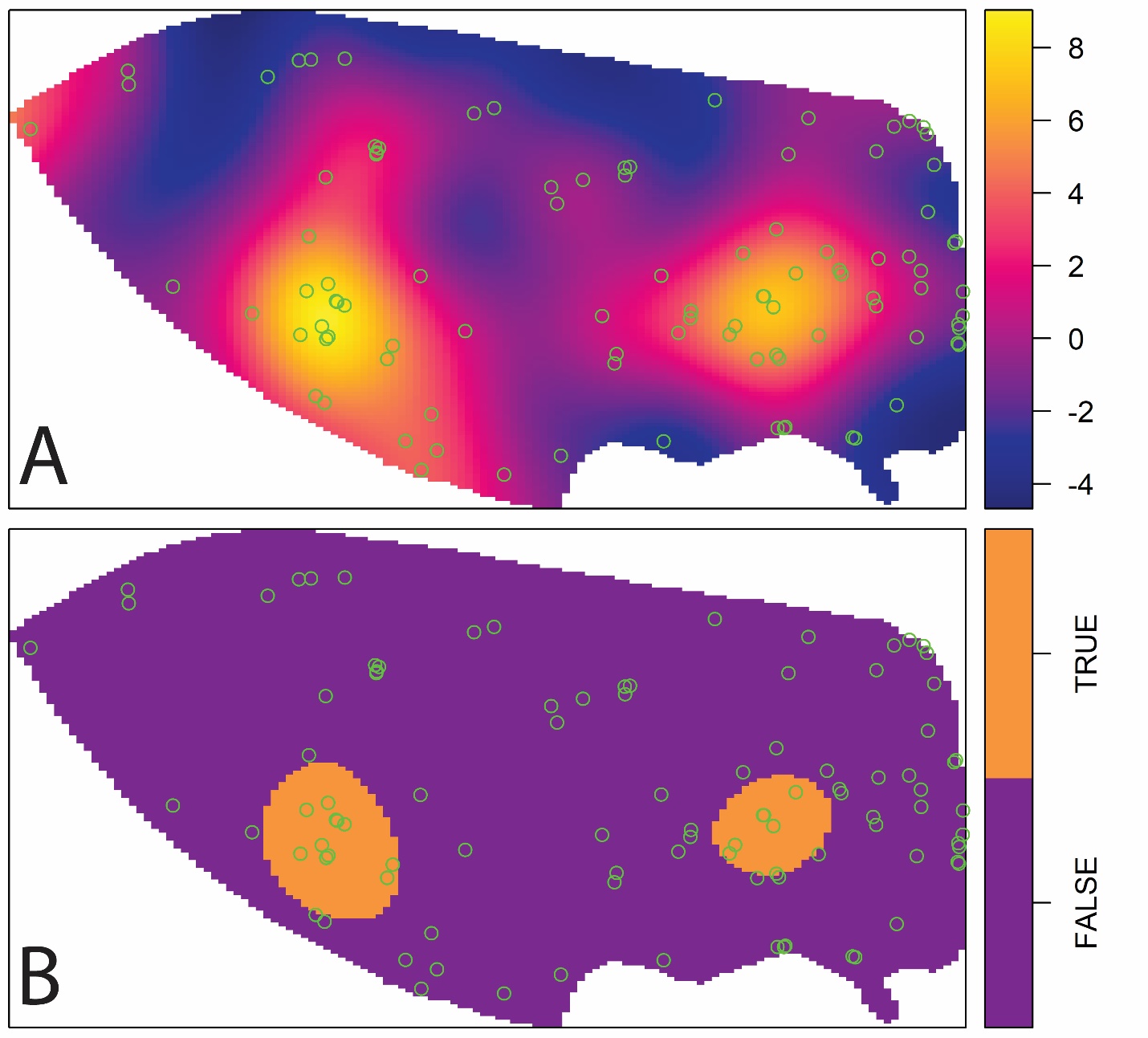
**

**Figure S3.** (A) Smoothed Pearson residual field for the inhomogeneous Poisson point process model containing third degree terms for both the *x* coordinate and distance from trails (*see* text for details). The ribbon on the right shows the corresponding color map for the values of the smoothed Pearson residuals. (B) Logical function showing areas where the absolute value of the smoothed Pearson residual field exceeded two standard deviations (= TRUE, in orange). Locations of *Chrysobalanus icaco* bushes (green dots) are superimposed on each surface. Overall, the model performed well, except for two subregions where counts were significantly higher than predicted.
